# Correction: Eroğlu, G. Electroencephalography-Based Neuroinflammation Diagnosis and Its Role in Learning Disabilities. *Diagnostics* 2025, *15*, 764

**DOI:** 10.3390/diagnostics15141772

**Published:** 2025-07-14

**Authors:** Günet Eroğlu

**Affiliations:** Computer Engineering Department, Engineering and Nature Faculty, Bahçeşehir University, 34349 Istanbul, Turkey; gunet.eroglu@bau.edu.tr

In the original publication [1], Figures 1, 2 and 4–7, as well as Tables 1 and 2, were based on earlier internal drafts and have since been revised for improved clarity and accuracy. While the overall methodology and conclusions of the study remain scientifically sound and unchanged, these updated figures and tables provide enhanced transparency and reproducibility of the presented data.

The corrected Figure 1, Figure 2, Figure 4, Figure 5, Figure 6, Figure 7 and Table 1, Table 2 are shown below.

Additionally, the Python code used for EEG preprocessing, Artificial Neural Network (ANN) model training, and performance evaluation was not included in the original publication. These scripts document key processes such as z-score normalization, data balancing, ANN architecture optimization, and performance metric calculation. To improve transparency and enable reproducibility, the code has now been made available as Supplementary File S1: Python codes MDPI.ipynb, and a citation for this file has been inserted in the Section 3 Results, paragraph 3, and should read as follows:

“Moreover, the study explored the impact of preprocessing techniques, such as minimum–maximum scaling, on model performance. While this technique introduced a slight reduction in accuracy, from 98.5% to 98.33%, the model retained robust performance metrics, including an F1 score of 0.983 and an increased loss of 0.08 (as detailed in Table 1 and Table 2). These findings indicate that while preprocessing can slightly alter accuracy, the overall diagnostic capability of the ANN remains consistently high (Supplementary File S1).”

With this correction, the “Supplementary Materials” part has been added accordingly.

**Supplementary Materials:** The following supporting information can be downloaded at: https://www.mdpi.com/article/10.3390/diagnostics15060764/s1, Supplementary File S1: Python codes MDPI.ipynb.

The authors state that the scientific conclusions are unaffected. This correction was approved by the Academic Editor. The original publication has also been updated.

## Figures and Tables

**Figure 1:**
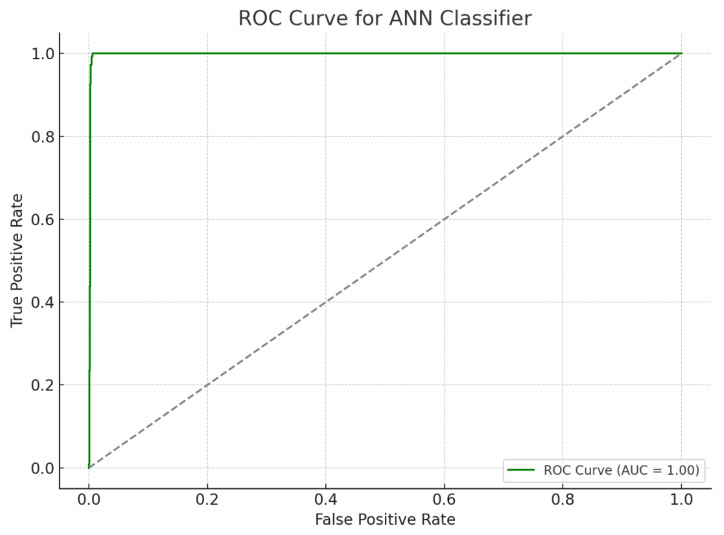
Receiver Operating Characteristic (ROC) Curve of the Artificial Neural Network (ANN) model. The ROC curve illustrates the performance of a multilayer perceptron model trained on EEG-derived features to classify children with learning disabilities. The model achieved an AUC of approximately 1, indicating high diagnostic accuracy in distinguishing between the LD and control groups. The gray dashed line in the ROC curve plot represents the “no-skill classifier” or random guess baseline.

**Figure 2:**
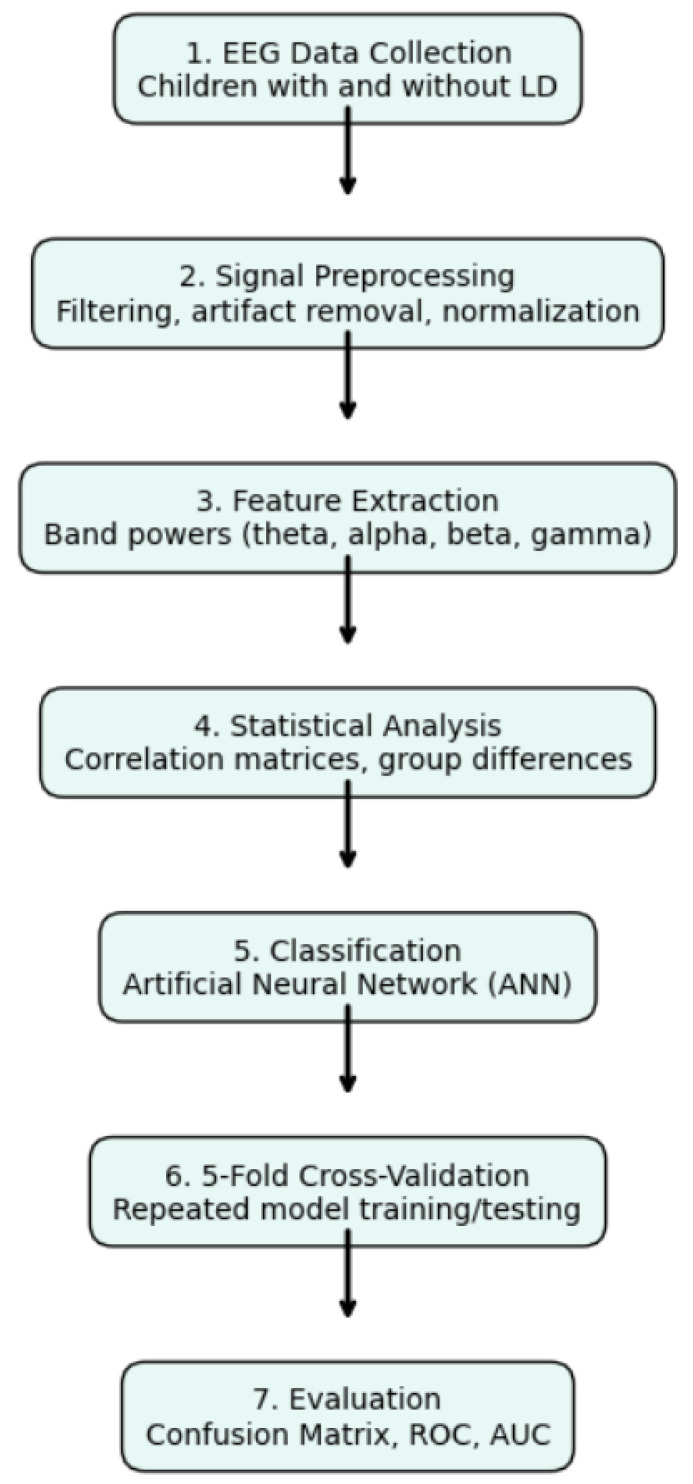
Stepwise flowchart of the EEG-based learning disorder diagnosis method. The figure presents a vertically aligned stepwise methodology for diagnosing learning disorders using EEG. The process includes data acquisition, signal processing, feature extraction, statistical comparison, 5-fold cross-validation, ANN-based classification, and performance evaluation.

**Figure 4:**
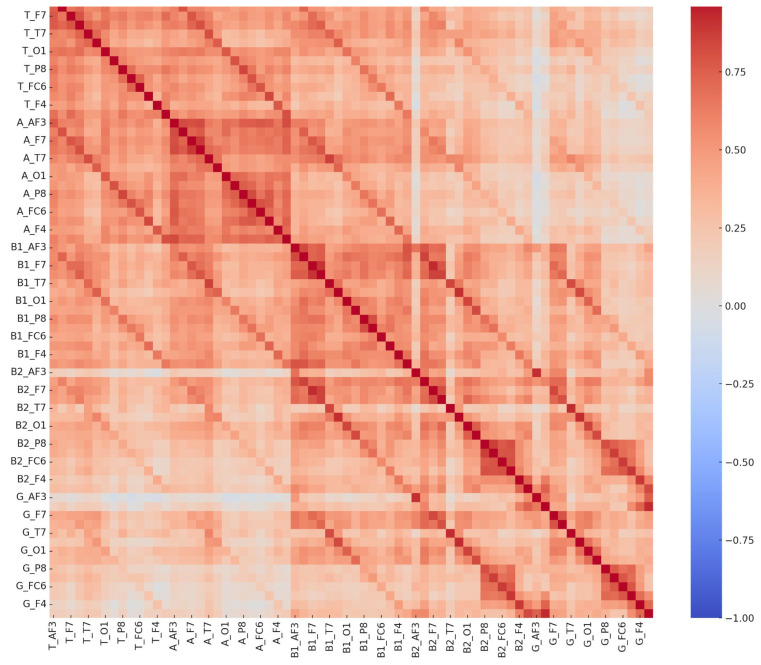
Comprehensive EEG channel correlation matrix—learning disorder group.

**Figure 5:**
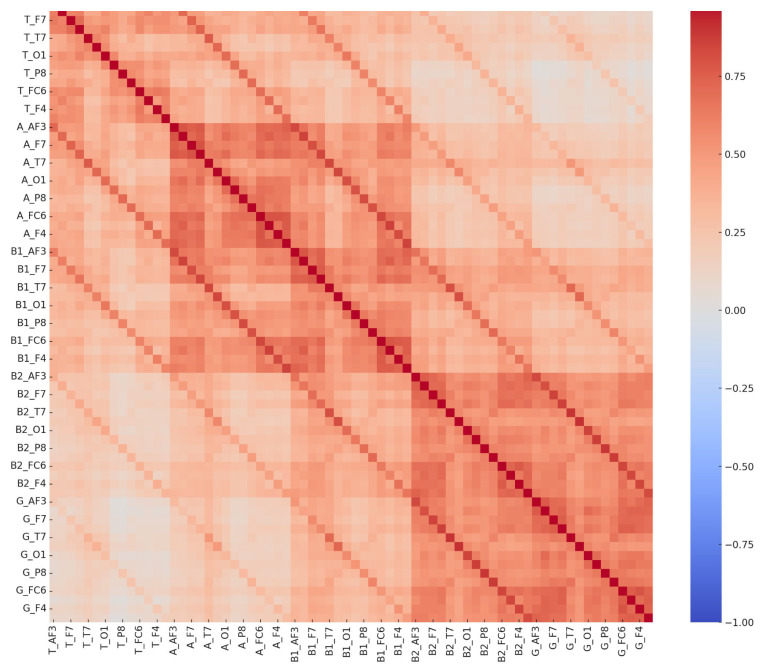
Comprehensive EEG channel correlation matrix—control group. These matrices depict interrelationships among 70 EEG-derived features in both the LD and control groups. The distinct correlation patterns reflect neurophysiological differences between groups, potentially supporting EEG-based biomarkers for learning disorder detection.

**Figure 6:**
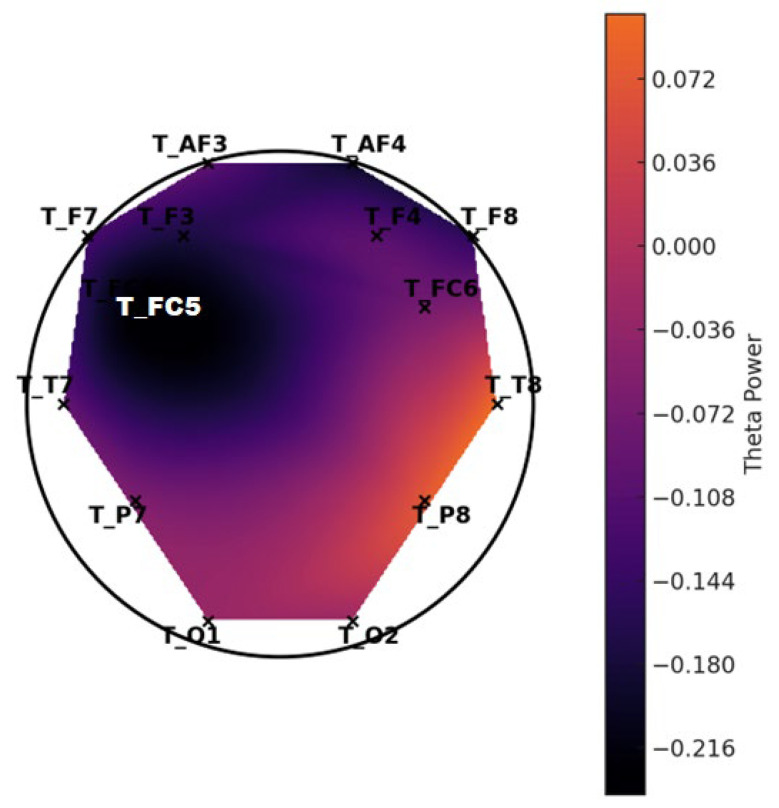
Topographic distribution of mean theta power—LD group.

**Figure 7:**
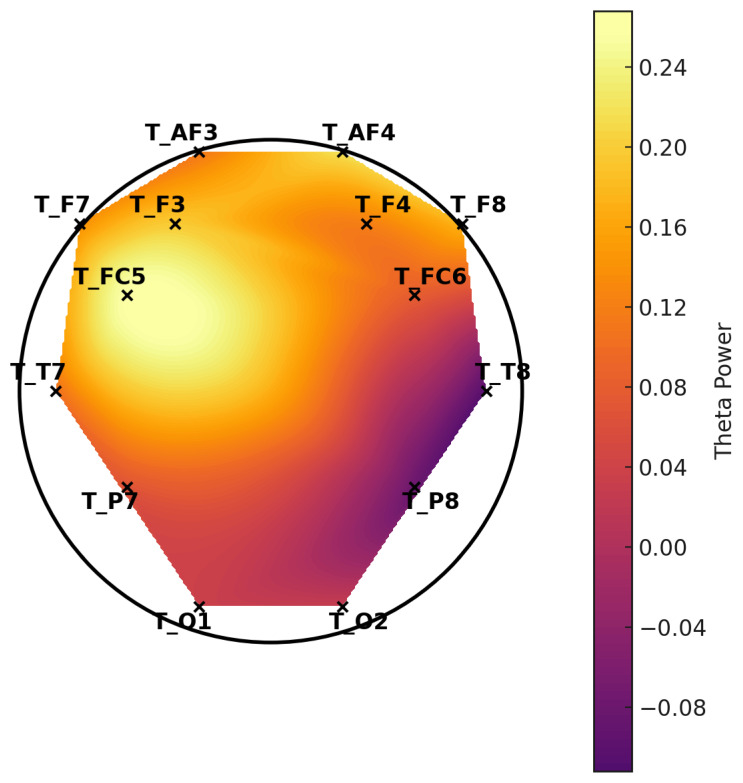
Topographic distribution of mean theta power—control group. These scalp maps represent the spatial distribution of average theta band power across selected EEG channels. The control group exhibits higher overall theta power, especially in the frontal regions, while the LD group shows relatively reduced frontal theta activity.

**Table 1:** Architecture of the Artificial Neural Network (ANN). The table outlines the structure of the ANN model used in the study.

Input Layer	70 EEG Features
Hidden Layer 1	64 neurons, Activation: ReLU
Hidden Layer 2	32 neurons, Activation: ReLU
Output Layer	1 neuron, Activation: Sigmoid
Total Layers	4

**Table 2:** Cross-validated ANN performance metrics (5-fold CV). This table presents the average performance metrics for the ANN model evaluated using 5-fold cross-validation.

Metric	Mean Value	Standard Deviation
Accuracy	99.49%	±0.0009
Precision	99.05%	±0.0017
Recall	100%	±0.0000
F1 Score	99.5%	±0.0008
Log Loss	0.031	±0.01
